# Management of a Rare Case of Multiple Coronary Artery Fistulas Associated with Ascending Aortic and Root Aneurysm: Case Report and Review of Literature

**DOI:** 10.3390/jcm13082297

**Published:** 2024-04-16

**Authors:** Mircea Robu, Bogdan Radulescu, Reza Nayyerani, Robert Enache, Ovidiu Stiru, Andrei Iosifescu, Georgiana Olaru, Raluca Ciomag (Ianula), Vlad Anton Iliescu, Horatiu Moldovan

**Affiliations:** 1 Faculty of Medicine, Carol Davila University of Medicine and Pharmacy, 050474 Bucharest, Romania; mircea.robu@drd.umfcd.ro (M.R.); reza.nayyerani@drd.umfcd.ro (R.N.); ovidiu.stiru@umfcd.ro (O.S.); iosifescuag@gmail.com (A.I.); raluca.ianula@umfcd.ro (R.C.); vladanton.iliescu@gmail.com (V.A.I.); horatiu.moldovan@umfcd.ro (H.M.); 2 Prof. Dr. C.C. Iliescu Emergency Institute for Cardiovascular Diseases, 022322 Bucharest, Romania; dr.georgianaolaru@yahoo.com; 3 Radiology Department, Fundeni Clinical Institute, 022322 Bucharest, Romania; robert.enache2006@yahoo.com; 4 Department of Cardiovascular Surgery, Emergency Clinical Hospital Bucharest, 014461 Bucharest, Romania; 5 Academy of Romanian Scientists, 050711 Bucharest, Romania

**Keywords:** coronary fistulas, aortic aneurysms

## Abstract

Coronary artery fistulas draining into the left ventricle is a rare finding. They can be associated with other congenital cardiac anomalies like ventricular septal defect or tetralogy of Fallot. While most of them are asymptomatic, they can lead to severe cardiac complications like infective endocarditis, heart failure, or myocardial ischemia. Symptomatic coronary artery fistulas can be managed surgically or percutaneously. We present a case of a 61-year-old male patient with both left anterior descending artery and right coronary artery fistulas draining into the left ventricle associated with ascending aorta and root aneurysm. Preoperative assessment for myocardial ischemia and the size and location of the fistulas was performed. The echocardiography stress test was negative. Surgery consisted of replacement of the ascending aorta and reconstruction of the noncoronary sinus with a Dacron patch with aortic valve preservation and no intervention for the coronary artery fistulas. The surgical strategy was adapted for cardioplegia administration to compensate for the volume of coronary blood drained into the left ventricle and for better protection of the distal myocardium.

## 1. Introduction

Congenital coronary artery fistulas (CAFs) are a direct communication of the epicardial coronary artery with one of the four cardiac chambers (coronary cameral fistulas—CCFs) or major vessels of the heart or chest (coronary arteriovenous fistulas) [1]. Incidence is 0.002% in the general population [2] and multiple coronary fistulas are found in 5% of cases [3]. CARs draining into the left ventricle are even more rare, most of them draining into the low-pressure right chambers [4]. Most of the CAFs are asymptomatic and can become symptomatic in the fifth or sixth decade of life [5]. While there is a diversity of nonspecific symptoms described in the literature like angina, dyspnea, palpitations, and syncope, CAFs can lead to more serious complications like myocardial ischemia, infective endocarditis, and heart failure [6,7,8]. When diagnosed, CAFs are usually solitary but can be associated with congenital cardiac anomalies like ventricular septal defect and tetralogy of Fallot [9]. Symptomatic CAFs can be managed either with direct surgical ligation with or without coronary bypass or with a variety of interventional methods (occluder devices, coils) [9].

We describe a rare case of multiple moderate CCFs, originating both from the left anterior descending artery and the right coronary artery and draining into the left ventricle, associated with ascending aorta and root aneurism.

To our knowledge, this association has not been reported in the literature. The case poses several questions for cardiac surgeons: what the correct surgical management of CAFs in this situation is, what is the correct dose of cardioplegia for optimal myocardial protection, and what kind of surgical technique should be chosen for aortic repair when considering the anatomy of the aortic root with aneurismal dilatation of coronary arteries.

## 2. Case Report

**Figure 1:**
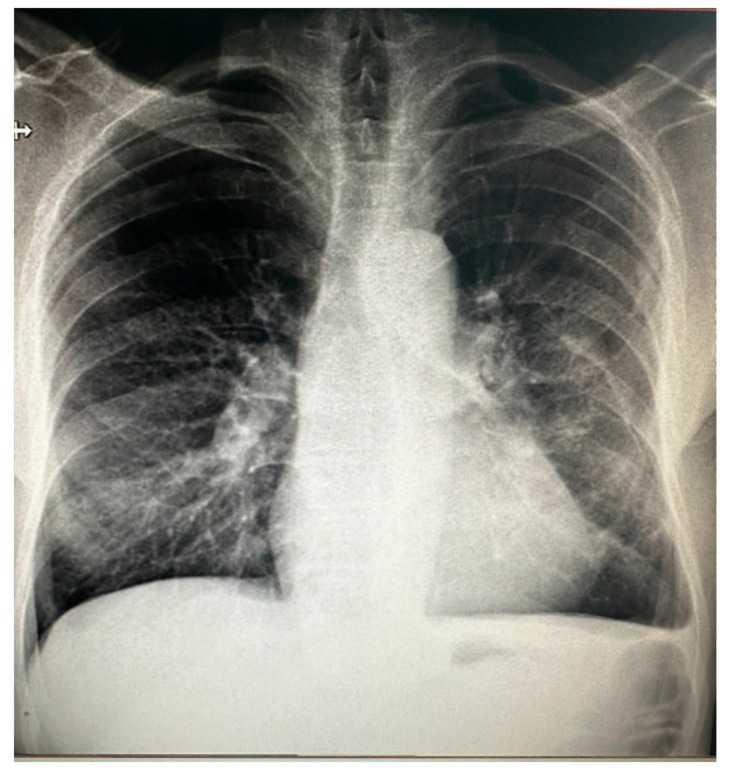
Posterior–anterior chest X-ray.

**Figure 2:**
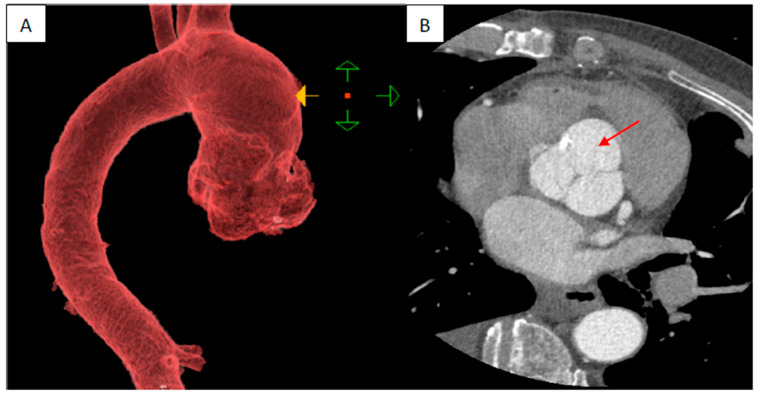
Preoperative contrast chest CT. (**A**) Reconstruction of ascending aorta and root. (**B**) The transverse plane at the level of the aortic sinuses shows an enlarged aortic root with the predominance of non-coronary sinus dilatation (arrow).

**Figure 3:**
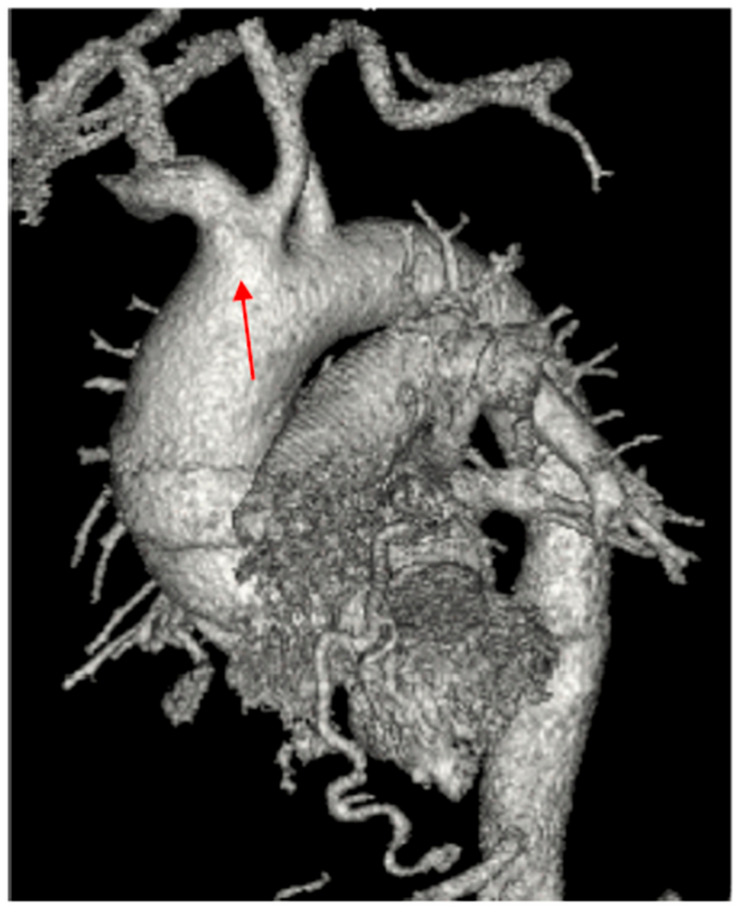
Contrast chest CT showing bovine aortic arch (arrow).

A 61-year-old male active smoker was referred to our center from a tertiary center for dilatation of the ascending aorta based on chest computed tomography (CT). His medical history was unremarkable, except for rare episodes of nonspecific thoracic pain. At admission, physical examination was within normal limits, he was hemodynamically stable with a systolic blood pressure of 120–140 mmHg on both hands and an 86-bpm regular pulse with normal peripheric pulses. Pulse oximetry revealed 98–99% oxygen saturation. EKG showed normal sinus rhythm with signs of left ventricular hypertrophy. Blood work showed only a mild renal dysfunction (serum creatinine 1.31 mg/dL, blood urea nitrogen 58 mg/dL). In a postero-anterior chest X-ray, the heart silhouette appeared enlarged, consistent with cardiomegaly, and both hilar regions appeared enlarged, with vascular appearance (Figure 1). Transthoracic echocardiography (TTE) showed the following diameters: aortic annulus of 26 mm, root diameter of 51 mm at the level of Valsalva sinuses, 54 mm for the ascending aorta, 44 mm aortic arch, and descending aorta of 35 mm. The left ventricle was concentrically hypertrophied with a systolic/diastolic diameter of 44/23 mm with normal global and regional function, the estimated left ventricular ejection fraction was 60%, and the right ventricle had a diameter of 27 mm and normal function. Aortic valve analysis showed a tricuspid aortic valve with mild regurgitation and mild mitral, tricuspid, and pulmonary valve regurgitations. No signs of aortic dissection were present, and no pericardial effusion was observed. Left and right atria were mildly dilated. Contrast chest CT (Figure 2A,B) measured the following diameters: aortic root of 60 mm at the level of aortic sinuses, asymmetric dilatation of the noncoronary sinus, 49 mm at the sino-tubular junction, ascending aorta of 57 mm, aortic arch 35 mm, and descending thoracic aorta 30 mm. A bovine aortic arch with the common origin of the innominate artery and left carotid artery was present (Figure 3). Also, the patient had signs of a recent right peripheral pulmonary embolism. A pneumological consult identified an older left peripheral pulmonary embolism and recommended only a follow-up chest CT at 3 months. To determine a possible cause of the pulmonary embolism, abdominal and pelvic contrast CT was obtained with no signs of neoplasia, or other pathological findings and upper gastrointestinal endoscopy was normal. Thrombophilia tests were performed: ATIII was 69% less than 83% normal value, APS was 1.22 slightly elevated, protein C and S had normal values, and lupus and anticardiolipin antibodies and anti beta2glicoprotein had normal values.

**Figure 4:**
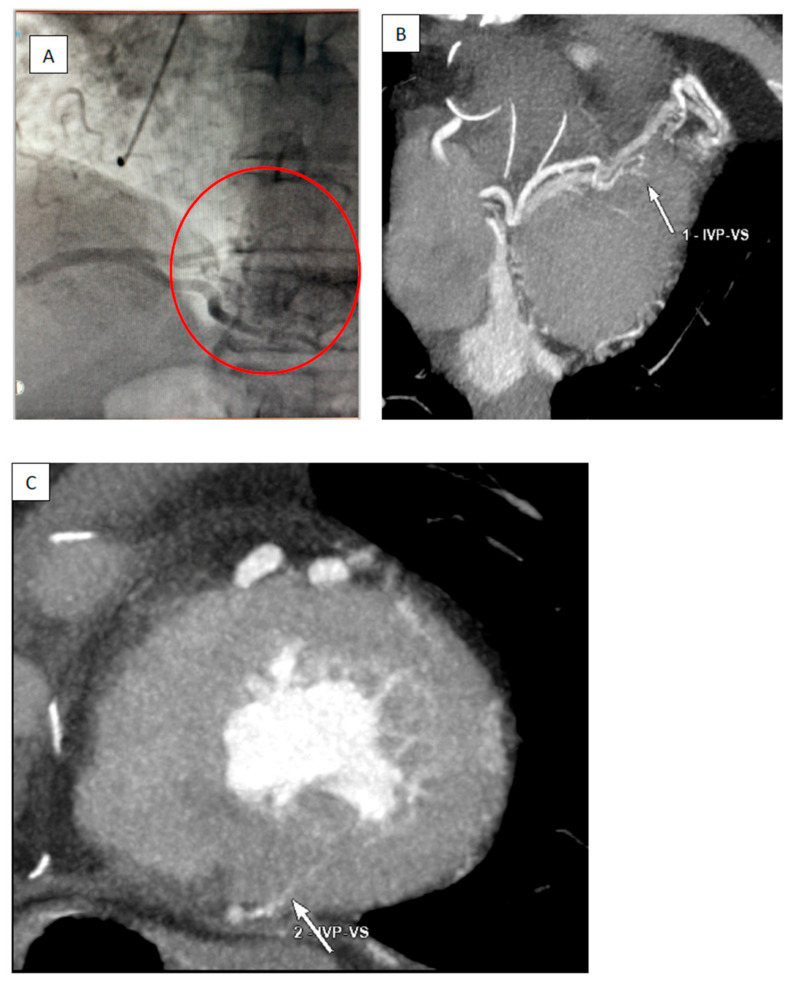
(**A**) Coronary angiography showing draining of the coronary contrast from the posterior interventricular artery into the left ventricle (circle insert). (**B**,**C**) Contrast chest CT showing coronary cameral fistula between posterior interventricular artery (IVP) and left ventricle (VS).

**Figure 5:**
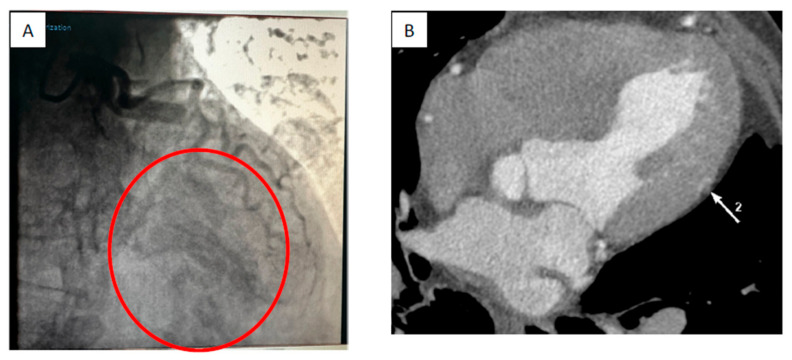
(**A**) Left coronary artery angiography showing drainage of contrast into the left ventricle from left anterior descending artery (circle insert); (**B**) contrast chest CT showing contrast into the left ventricle (arrow) from the left anterior descending artery.

Coronary angiography was performed with no significant coronary artery stenosis. However, the left anterior descending artery (LAD) and right coronary arteries (RCAs) were dilated, and two small to medium coronary–cameral fistulas were described between the distal LAD and the left ventricular cavity and between the posterior interventricular artery and the left ventricular cavity (Figure 4A–C and Figure 5A,B). To investigate possible “myocardial steal” and myocardial ischemia related to CCFs, a stress echocardiography test was performed with a negative result for myocardial ischemia. Doppler ultrasound of carotid arteries and femoral arteries was normal.

### Surgical Technique

**Figure 6:**
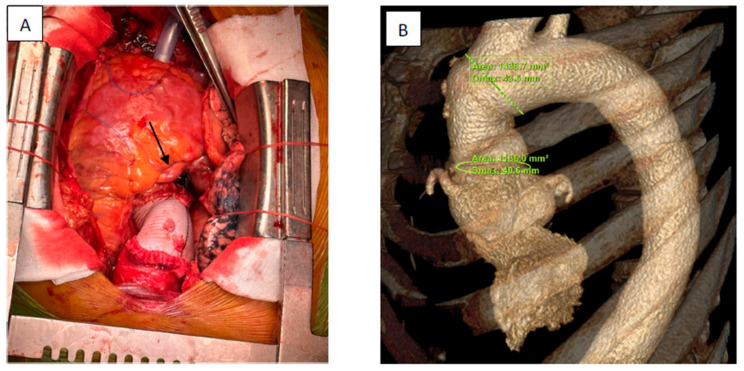
(**A**) Intraoperative view showing ascending aorta replacement and reconstruction of the non-coronary sinus with a Dacron patch and a dilated right coronary artery (arrow); (**B**) postoperative contrast chest CT reconstruction of the ascending aorta with a 40.6 mm diameter of the ascending aorta at the level of the sino-tubular junction and a 43.6 mm diameter of the proximal aortic arch.

After general anesthesia, a median sternotomy was performed. Ascending aorta and root aneurism were observed with an asymmetric dilatation of the aortic root especially at the level of the noncoronary sinus. The distal portion of the ascending aorta and aortic arch were normal. A 23 Fr cannula was inserted into the aortic arch and a two-stage venous cannula was inserted into the inferior vena cava via the right auricle. A cannula was inserted into the coronary sinus for retrograde cardioplegia. A vent was inserted via the right superior pulmonary artery into the left ventricle. Cardiopulmonary bypass was started, and the heart was arrested with cold blood cardioplegia (Del Nido). The patient received 1500 mL of cardioplegia with 500 mL more than the initial calculated dose to compensate for the coronary cameral fistulas. First, we administered 500 mL of cardioplegia in the retrograde fashion, and then the remaining dose selectively via the coronary ostia after aortotomy. A dose of 500 mL was added to the initial calculated dose of cardioplegia after we had quantified the volume of cardioplegia drained into the left ventricle based on data from the vent cannula. The patient had a normal tricuspid aortic valve with traces of aortic regurgitation on preoperative TTE. We decided to preserve the native aortic valve and we replaced the ascending aorta and reconstructed the noncoronary sinus. Because no signs of myocardial ischemia were observed preoperatively, we did not consider it necessary to close the CCFs. We excised the noncoronary sinus, preserving 5 mm of aortic wall, and a new sinus was tailored using a patch of Dacron prosthesis. The patch was sewn in place using a 4.0 Polypropylene continuous suture beginning from the nadir of the sinus and continuing both sides until the commissure level was reached. The ascending aorta was excised from 5 mm above the sino-tubular junction up to 1 cm distal to the innominate artery origin. A 34 mm Dacron prosthesis was used to replace the portion of the ascending aorta (Figure 6). The heart was de-aired, the aorta unclamped, and CPB was weened without any incidents. The operation was concluded in the usual fashion. Intraoperative transesophageal echocardiography showed traces of aortic, mitral, and tricuspid regurgitation with good biventricular function. Cardiopulmonary bypass time was 143 min and aortic cross clamp time was 88 min.

Postoperative evolution was favorable, and the intensive care stay was 2 days. The patient was extubated 3 h after surgery without neurological deficits. He was hemodynamically stable with minimum vasopressor medication requirements on the first day after surgery. Chest drains were removed on the second day after surgery. No blood transfusions were necessary during or after surgery. We did not observe any signs of myocardial ischemia in the postoperative setting. Serial EKG showed normal sinus rhythm with the same left ventricular hypertrophy pattern. CK/CK-MB evolution was the following: 314/64 U/L before surgery, 665/61 U/L on postoperative day one, 687/43 U/L on the second day after surgery, and 572/32 U/L on the third day after surgery, with a continuous descending trend. Daily TTE showed normal global and regional function, traces of mitral, aortic, and tricuspid regurgitation, and no pericardial or pleural effusion. The only postoperative complication was a new onset of rapid atrial fibrillation on day one without hemodynamic impact, converted to sinus rhythm with Amiodarone. After transfer to the surgery ward, the evolution was unremarkable, and the patient was discharged on day 7 after surgery. Follow-up at 1 month, 3 months, 6 months, and one year with serial transthoracic echocardiograms showed traces of aortic regurgitations, no progression of the diameter of the aortic root, and a good function of the heart. The patient remained asymptomatic with good effort tolerance.

## 3. Discussion

To our knowledge, the association between CAFs and aortic aneurysms has not been reported in the literature. Also, CAFs draining into the left ventricle is a rare situation. Several questions for cardiac surgeons arose regarding the correct surgical management of the CAFs, what dose of cardioplegia should be administrated for optimal myocardial protection, and what kind of surgical technique should be chosen for aortic repair considering the anatomy of the patient.

We decided not to intervene at the level of the CAFs. This decision was made based on several reasons. First, ACC/2008 guidelines for the management of adults with congenital heart disease recommend not intervening in asymptomatic small to moderate CAFs [10]. In our case, the CAFs were classified as small–moderate, they were distally located, and they were asymptomatic based on clinical assessment and a negative stress test for ischemia. Second, the relative distal locations of the CAFs favored future interventional management if the patient would be symptomatic in the 7th decade.

We opted to use cold blood cardioplegia (DelNido) for several reasons. First, in the literature, multiple reports communicate better outcomes with DelNido cardioplegia in terms of better preservation of cardiac index, better preservation of myofibrillar, and decreased morbidity [11]. Second, from a technical point of view, we estimated that a 90 min aortic clamp time from a single dose of DelNido cardioplegia was sufficient for aortic repair. The most important decision remained the optimal dose of cardioplegia, considering that we could not quantify the amount of cardioplegia drained into the left ventricle during administration. Also, we had to consider the higher mass of the myocardium because of left ventricle hypertrophy. We calculated the theoretical dose of DelNido based on current recommendations: 20 mL/kg, no more than 1000 mL in an adult over 50 kg [11]. Our patient weighed 80 kg so the theoretical dose was 1600 mL. According to the protocol, the initial dose of cardioplegia was set to be 1000 mL. To compensate for the volume of cardioplegia drained into the left ventricle, we measured the volume of blood aspirated by the vent cannula during cardioplegia administration. This volume was approximately 500 mL, and we supplemented this volume. In total, 1500 mL of cardioplegia was administered. Also, this dose was administrated both in the anterograde and retrograde fashion for better myocardial protection.

Regarding surgical technique, we opted for reconstruction of the non-coronary sinus and ascending aorta replacement with a Dacron graft. We chose this technique for several reasons. First, the aortic valve was tricuspid and functioned normally, so a valve-sparing procedure was appropriate. We estimated that replacing only the non-coronary sinus and the ascending aorta required less aortic cross-clamp time than root replacement. The need for shorter aortic cross-clamp time, besides the fact that it is universally better in all patients, was important in our context for better myocardial protection considering that a volume of cardioplegia was lost into the left ventricle. Second, based on preoperative contrast CT images, the aortic root was asymmetrically dilated, especially at the level of the non-coronary sinus, so it made sense to reduce operative times by only replacing this part of the aortic root. Third, we considered that the aneurismal dilatation of both the left and right coronary arteries especially at the origin could compromise coronary reimplantation into the prosthesis because of the fragility of the coronary wall.

## 4. Review of Literature

Congenital coronary artery fistulas, first described in 1865 by Krause, are a rare finding. They comprise 0.8% of all coronary anomalies and have become more detectable in recent years because of advances in cardiac diagnostic imaging, with an incidence of 0.1% in all cardiac catheterization patients [2]. The right coronary artery (RCA) is the most common site of origin of CAFs, in 50% of patients. Other sites include the left anterior descending artery (LAD) in 35% to 40%, the left circumflex artery (CxA) in 5% to 20%, and multiple coronary arteries in 5% [3]. A study of 6341 patients diagnosed CAFs using coronary CT angiography [10]. The authors described the following CAFs: coronary artery to pulmonary artery was most frequent with an incidence of 76.8%, coronary to bronchial artery in 8.9%, coronary to cardiac chamber in 8.9%, combined coronary to pulmonary artery and coronary to bronchial artery in 3.6%, and coronary to superior vena cava in 1.8%.

Coronary cameral fistulas (CCF) are a rare finding with most of the fistulas draining to the lower-pressure right side of the heart rather than to the high-pressure left side [4]. They can be explained by the persistence of embryonic intratrabecular spaces and sinusoids [3]. In the study mentioned above, only five cases were diagnosed. Four of them originated from the RCA and one from the LAD artery. Drainage sites were left ventricle in two cases and left atrium in the remaining cases. Patients with CCFs are younger than patients with coronary venous fistulas, with no significant differences regarding gender or aneurysmal formation, rupture, or pericardial effusion [6,12]. Drainage into the left or right ventricle is rare [6]. Said et al. reported that few LAD fistulas ended in the left or right ventricle, and no data are reported for the CxA fistulas, while only 12% of RCA fistulas drain in the left ventricle [8].

The long-term implications of CAFs are still a matter of debate. While the consensus is that most CAFs are asymptomatic, in some cases, failure to recognize them leads to incorrect diagnosis and altered treatment. Recent data suggest that CAFs remain asymptomatic in the first two decades and more than two-thirds become symptomatic in the fifth and sixth decades of life [5]. Clinical presentations of LAD fistulas are angina [7], dyspnea [13], fatigue [14], palpitations [15], syncope [12], cardiac heart failure [16], and infective endocarditis [17]. For the RCA fistulas, presentation for chest pain [18], dyspnea [19], palpitations [20], and syncope [21] are reported. Severe complications like infective endocarditis and myocardial infarction are relatively uncommon and occur more frequently in males. Infective endocarditis is reported exclusively in CCFs with unilateral or bilateral origin and can be explained by the higher turbulent flow and endothelial damage in a dilated chamber compared with a vascular structure [6]. Myocardial infarction seems to be associated with coronary venous fistulas and coronary steal phenomenon with impaired distal perfusion [7]. Cardiac heart failure seems to be more frequent in female gender and unilateral fistulas are predominant [12]. Most of the cases of patients with cardiac heart failure involved coronary to arterial pulmonary fistulas with dilated cardiomyopathy and pulmonary hypertension [22].

While up to 80% of CAFs are isolated, various cardiac conditions can be associated. The most common congenital anomalies associated are ventricular septal defect, tetralogy of Fallot, patent ductus arteriosus, atrial septal defect, and pulmonary atresia with an intact ventricular septum [9]. There are reports of coronary artery disease associated with CAFs and lung parenchymal abnormalities such as bronchiectasis of hypoplasia of the pulmonary vasculature [10]. Atherosclerotic coronary artery aneurysms associated with CAFs are described as rare and so far only 50 cases have been reported in the literature, while simple aneurysmal dilatation of the coronary fistulae is also described [23]. Described complications of atherosclerotic coronary aneurysms are rupture of the aneurysm, thrombus propagation, and distal emboli [24]. We could not find any data regarding an association between ascending aortic aneurysms and CCFs.

Diagnosis of CAFs includes coronary angiography, CT coronary angiography, and echocardiography. Coronary angiography is traditionally the gold standard investigation. It enables precise visualization of the anatomy, the origin, and the insertion of fine vessels and other associated structural defects and information regarding hemodynamics [25]. Transthoracic and trans-esophageal echocardiography provide information regarding pressure estimation in cases of shunt and delineating the flow in the aneurysmal artery or recipient chambers [26]. An important role of echocardiography is detecting other associated acquired or congenital cardiac defects, masses, or thrombotic lesions. CT coronary angiography has become the gold standard diagnostic evaluation tool for CAFs because of the higher temporal and spatial resolution and for the possibility of 3D reconstruction and multiplanar slices. The CAF’s origin, course, and drainage can be examined [3]. Myocardial perfusion imaging with nuclear testing helps detect and quantify myocardial ischemia [27]. Intravascular ultrasound also has a role in assessing the flow dynamics, delineating clots in fistulous tracts, thrombus, and aneurysmal segments, and measuring vessel size for therapeutic reasons [28]. In our case, the best visualization of CAF and coronary anatomy was with contrast chest CT. Because the patient’s fistulas opened in the high-pressure left ventricle, with contrast rapidly dissipating, coronary angiography images were less helpful in visualizing the CAF and had only a diagnostic purpose.

Management of CAF depends upon its origin, drainage, size, symptoms, and associated cardiovascular abnormalities [2]. While asymptomatic patients do not require any treatment, large- and moderate-size CAFs with symptoms such as arrhythmia, ventricular dysfunction, or myocardial ischemia have a class 1 recommendation for closure according to European Society of Cardiology and American Heart Association guidelines [2]. Initially, surgical treatment was the first choice with a perioperative mortality of 2 to 4% and a 3.6% risk of myocardial infarction [29]. There are two techniques for definitive closure: the “epicardial approach”, consisting of external ligation with or without coronary artery bypass distally, and the “endocardial approach” in which direct suture is performed from within the recipient chamber [24]. The main advantage of surgery is that it is associated with a reduced risk of fistula recurrence, especially with the “endocardial approach” [30]. Percutaneous transcatheter treatment is an alternative to surgery and various percutaneous techniques are described: vascular plugs, covered stents, umbrella devices, detachable balloons, coils, and ductal occluders [2,25]. Percutaneous management depends on the site of the CAF. In proximal CAFs, percutaneous closure is generally performed at the proximal end or extremely distally to the drainage site. For distal fistulas, an occluder device is placed as distally as possible so that the flow of the proximal artery is not compromised [3]. Failure of the percutaneous procedure can be observed in large CAFs. Also, distal coil migration, incomplete closure, and complete closure of the native vessel blood supply with subsequent myocardial ischemia are possible [27].

## 5. Conclusions

We have presented a rare association of multiple CAFs (left anterior descending artery and right coronary artery draining into the left ventricle cavity) and ascending aorta and root aneurism managed surgically, without any intervention necessary at the level of the fistulas. Careful preoperative assessment of the CCFs regarding the “coronary steal” phenomenon, possible myocardial ischemia, and their size and location should be performed. Preoperative myocardial ischemia should determine that the surgeon surgically ligates the fistulas with or without coronary bypass in the same setting, while the dimension and size of the fistulas determine the cardioplegia administration strategy. Our strategy was to compensate for the volume of blood drained into the left ventricle by supplementing the dose of cardioplegia delivered in an anterograde and retrograde fashion for better myocardial protection.

## Data Availability

Data available on request.
